# Parental Stress and Satisfaction in Parents With Pre-school and School Age Children

**DOI:** 10.3389/fpsyg.2021.683117

**Published:** 2021-05-28

**Authors:** María de los Angeles Oyarzún-Farías, Félix Cova, Claudio Bustos Navarrete

**Affiliations:** ^1^Departamento de Psicología, Facultad de Ciencias Sociales, Universidad de Concepción, Concepción, Chile; ^2^Escuela de Trabajo Social, Facultad de Ciencias Sociales y Comunicaciones, Universidad Santo Tomás, Concepción, Chile

**Keywords:** parenting, parents, parental stress, parental satisfaction, gender

## Abstract

Parenting is a transforming experience for the life of parents that brings joy and satisfaction as well as challenges, frustration, and demands. The aim of this study was to determine the relationship between “parental stress and satisfaction” and work-home conflict, perceived social support, and global satisfaction with life, and to determine the moderating role of the parent's gender. A sample of 244 participants was studied: 49.6% (121) mothers and 50.4% (123) fathers with children between 2 and 12 years of age. The data was analysed by means of multiple linear regression models and Beta regression for stress and parental satisfaction, respectively, and they were complemented with general and conditional dominance analyses to estimate the relevance of the predictors. Mothers presented higher levels of parental stress and satisfaction than fathers. Being female, having two children, and home-work conflict were predictors of a higher parental stress. Age was also a predictor, as younger parents and also those older than 37 years of age showed more parental stress. Having no partner and being male was associated to lower parental satisfaction. In conclusion, parental stress and satisfaction emerge as clearly differentiated dimensions of parenting experience. The gender gap in parental stress could be linked to the persistence of traditional roles regarding the care of the children, in agreement with the findings in other research.

## Introduction

Being a parent implies both a role as an identity and as well as a subjective experience (Bornstein, 2015). In general, parenting is a transforming experience (Nelson et al., 2014; Bornstein, 2015). Usually, it contributes to joys and satisfactions of great meaning for people's lives; it also implies facing a series of challenges and demands that imply less publicly recognised negative emotions such as frustrations, oppression, and fear (Nomaguchi and Milkie, 2003; Nelson et al., 2014; Bornstein, 2015; Kohler and Mencarini, 2016). The term “parenting paradox” has been coined to recognise these opposing trends in the experience of being a parent (Rizzo et al., 2013; Erez and Shenkman, 2016; Louie et al., 2017; Nomaguchi and Milkie, 2020), which usually provides meanings and benefits, but which also implies costs and discomforts (Nomaguchi and Milkie, 2003; Umberson et al., 2010; Musick et al., 2016).

There is growing interest in identifying what factors are associated with higher degrees of well-being in parents or, on the contrary, with more negative experiences and emotions. This study is focused on two specific aspects: the feelings of demand imposed by the role of parent, referred to by the concept of parental stress; and on parental satisfaction, related to the positive feelings that arise in the parents themselves in the exercise of their roles (Oronoz et al., 2007).

The stress and satisfaction that parenting can imply are influenced by various factors. One factor that requires detailed analysis is gender. Various studies show that fathers experience greater satisfaction with life and happiness than mothers, and that they live with more satisfaction and less stress their parental role (Nomaguchi and Milkie, 2003; Aassve et al., 2012; Hansen, 2012; Nelson et al., 2013, 2014; Nelson-Coffey et al., 2019). These findings are not surprising if we consider that it is usually mothers who assume a primary role in the care of children and in the most demanding tasks in relation to parenting, as well as greater domestic responsibilities in general. Mothers tend to experience greater time pressure than fathers as early as in the transition to parenthood (Ruppanner et al., 2019). Nevertheless, recent investigations show a certain trend to a decrease in the in the gender gap regarding the costs and rewards associated to parenting (Nomaguchi and Milkie, 2020). The parental role also implies its particular stressors; in particular, the traditional role of provider is very demanding for the fathers who have difficulties to obtain sufficient economic resources to cover the needs of their family group (Barnett and Gareis, 2009; Boyraz and Sayger, 2009; Vuori and Astedt-Kurki, 2013).

Another factor that can affect parental stress and satisfaction is work-home and home-work conflicts. The home-work and work-home conflict refer to “a sort of conflict of roles in which the pressures from work and from the family are mutually incompatible in some aspect” (Moreno et al., 2009) and which is characterised by its bidirectionality. Though, initially, the attention was focused on the interferences of work demands in family life (work-home conflict), it was later broadened to include the interferences of family life demands in working life (home-work conflict).

Interest in this issue emerged, largely, because of the implications of the growing incorporation of women into the labour market, and it has been focused on how work demands can hinder family life, increase childcare stress, and reduce life satisfaction at various levels (Kulik and Liberman, 2013; Kulik et al., 2015; Caro et al., 2017). However, attention has been drawn to the other possible direction of this relationship: the way in which the demands and conflicts of family life can affect, in turn, working life and, hence, increase stress and affect people's well-being (Moreno et al., 2009). The initial focus on work-home and home-work conflicts in mothers has also expanded to consider how these conflicts can affect fathers (Pereira et al., 2017). This has been stimulated by the changes that have taken place in the ways of understanding and living parenthood, where the figure of the providing and distant father tends to be replaced by that of a father who is more present and involved in parenting (Kulik et al., 2015; Aguayo et al., 2016).

In contemporary societies, people have often seen the broadest social and family networks of belonging (Arriagada, 2004; Perilla and Zapata, 2009). The upbringing of children has become an activity that parents often carry out with little accompaniment of family and community networks (Rochlen et al., 2008; Pollmann-Schult, 2018). Studies suggest that the perception of greater support from others in the development of parenting tasks would be associated with more positive emotions and less stress from parenting (Nelson et al., 2014).

The purpose of this study was to determine the stress and parental satisfaction of parents and also the relationship of these two poles of parental experience with the work-home and home-work conflict, as well as with perceived social support, and with a more unique aspect of each person such as their overall level of satisfaction with life. Various sociodemographic factors that can positively or negatively impact the balance between the costs and rewards of the parental experience were considered, such as socioeconomic status, partnership status, parents' age, and children's age. Since parenting has its own particularities depending on the children's developmental stage (Nelson et al., 2014; Nomaguchi and Milkie, 2020), the scope was narrowed down in this study to an age range: parents with children between 2 and 12 years of age. A specific objective was to determine the possible moderating role of the parent's gender in the relationships considered.

## Materials and Methodology

### Participants

The participants were selected by means of a stratified sample. The area/territory selected for this study was made up of 36 communes of Greater Santiago, Chile. Six groups were created from the total number of communes taking into consideration autonomous income median, educational level, and proportion of multidimensional poverty, and two communes from each of the six groups were selected. The city blocks and homes were selected at random. However, because of accessibility reasons we had to use snowball sampling in the upper-middle socioeconomic level.

The study considers two overlapping samples. The first sample is made up of 352 parents with at least one child between 2 to 12 years of age. In this sample, only the psychometric analysis of the Scale of Parental Stress was carried out (Oronoz et al., 2007). The other analyses were carried out in the second sample, made up of the subgroup of parents where all of their children were between 2 to 12 years of age, which corresponded to 244 participants. The participants were made up of 49.6% (121) mothers and 50.4% (123) fathers. The average age was 33 years old (SD = 6.6; from 20 to 46 years old), with an average of 1.4 children (SD = 0.64; from 1 to 4 children); 68.4% (167) had 1 child; 25% (60) 2 children; 6.1% (15) 3 children, and 0.8% (2) 4 or more children. Regarding the children's age, 46.7% (114) had children in pre-school age, 34% (82) in school age, and 20% (48) in pre-school and school age.

Regarding the participants' partnership status, 57% (138) were in the category “*with a partner*” (married, civil union, de facto relationship), 14% (34) “*other partnership*” (divorced, separated de facto, single, widowed), and 29% (72) “*no partner*.”

Regarding the socioeconomic status (SES), 5% (11) ranged from a “very high” SES (A), 13% (29) “upper” SES (B), 25% (55) SES “upper-middle” (Ca), 26% (58) “middle” SES (Cb), 26% (58) “lower-middle” SES (D), and 5% (12) “low” SES (E).

### Instruments

#### Parental Stress Scale (Oronoz et al., 2007)

Spanish adaptation of the Parental Stress Scale-PSS (Berry and Jones, 1995). The original scale has 18 items that describe the parent-child relationship and how the parent feels regarding their parental role. It includes four factors: parental rewards, parental satisfaction, parental lack of control, and parental stressors. In this investigation, the Spanish 12-item version was used (Oronoz et al., 2007). The study this version was derived from observed two factors that represent the positive and negative aspects of the parenting. One factor was named “*baby rewards*” (because of the age of the children included in the study which ranged from 3 to 8 months of age), which refers to the degree of satisfaction that parents experience in the exercise of their parental role. The other factor was named “*parental stressors*,” which refers to the levels of demand that parents perceive as derived from their parental roles. An item example of each factor is “I feel happy in my role as a parent” and “I feel overwhelmed by the responsibility of being a parent,” respectively. The alpha value of each factor was 0.76 for stressors and 0.77 for baby rewards (Oronoz et al., 2007).

As shown in the results section, the confirmatory factor analysis of the scale carried out in this study showed adequate adjustment levels for the model with the same factors and items of the Spanish version. Because of the confusing name of the subscale “baby rewards,” and considering the participant's children's age of this investigation, it was deemed appropriate to rename it as “*parental satisfaction*” in agreement with the aspect it measures.

The instrument presented adequate psychometric properties with an alpha value of 0.88, CI 95% [0.77, 0.93] for the parental satisfaction subscale and 0.85, CI 95% [0.82, 0.88] for the parental stress subscale. The coefficient Omega, suggested by some authors as a better measurement of internal consistency (Dunn et al., 2014; Peters, 2014; Ventura-León and Caycho-Rodríguez, 2017), had analogue values: 0.88, CI 95% [0.81, 0.95] and 0.85, 95 CI% [0.82, 0.88], respectively.

#### Survey Work-Home Interaction (SWING)

The original Survey Work-Home Interaction-Nijmegen was created by Geurts et al. (2005). There is a Spanish version of 22 items distributed in four subscales: negative work-home interaction, negative home-work interaction, positive work-home interaction, and positive home-work interaction. This version has shown adequate psychometric properties (Moreno et al., 2009). The questions are presented in four-option Likert format with scores between 0 (never) and 3 (always). In this study, the two subscales of negative interaction were used: work-home conflict, which includes eight items (e.g., Is it difficult for you to take care of your domestic obligations because you are constantly thinking about your work?); and home-work conflict, made up of four items (e.g., Is difficult for you to concentrate on your work because you are worried about domestic issues?).

Both scales showed suitable internal consistency. The alpha and omega values for each subscale were the following: 0.88, CI 95% [0.85, 0.91] and 0.89, CI 95% [0.86, 0.92]; 0.76, CI 95% [0.63, 0.83] and 0.77, CI 95% [0.67, 0.86], respectively.

#### Perceived Social Support

In order to measure social support, an *ad hoc*. scale was created. The participants were asked to evaluate in a five-option Likert scale (1 = never; 5 = always) the frequency of the following types of support when they needed them: somebody to spend a good time with; somebody to trust or to talk to about yourself and your concerns; somebody to love and to make you feel loved. The items were selected from the validated Spanish version of the MOS Social Support Survey (De La Revilla et al., 2005), because due to time constraints using the whole survey was not possible. The alpha value was 0.78, CI 95% [0.68, 0.84] and the omega value was 0.79, CI 95% [0.71, 0.86].

#### Satisfaction With Life Scale SWLS

This scale includes five closed questions focused on satisfaction with life (Diener et al., 1985). The questions were presented in a seven-option Likert format that ranged from 1 (totally disagree) to 7 (totally agree). A question example is: *Am I satisfied with my life?* This scale is a tool widely used internationally (Diener et al., 2013; Nelson-Coffey et al., 2019). Its use in the Chilean adult population has shown adequate psychometric properties (Vera-Villarroel et al., 2012). The alpha value observed in this study was 0.85, CI 95% [0.80, 0.88] and the omega value was 0.85, CI 95% [0.81, 0.89].

#### Sociodemographic and Family History Questionnaire

Information was collected about gender, socioeconomic status, parent's age, number of children, children's age, and partnership status. In order to estimate the socioeconomic status, the adapted ESOMAR (Adimark, 2000) was used, which establishes the family socioeconomic status from the educational level and occupational category of the main family provider, which produces six levels from “very high” SES (A) to “low” SES (E).

In order to include children's age in a single variable, three categories were created: (a) preschool-age child, (b) school-age child, and (c) both (preschool and school-age child). The partnership status variable was obtained from the combination of two variables: marital status measured in seven options (married, civil union, de facto relationship, divorced, separated de facto, single, and widowed) and the dichotomous question “present partnership,” which created three categories for analysis: (a) “*with partner*” (married, civil union, de facto relationship); (b) “*other partner*” (divorced, de facto separated, single, and widowed); and (c) “*no partner*.”

### Data Collection Procedure

The instruments were applied at the participant's home. Each interview lasted ~45 min. The correct conduction of the interviews was audited in 5% of the participants.

The research was approved by the Ethics Committee of the University of Concepción, Chile. Mothers and fathers provided written informed consent prior to their participation and all data was used anonymously and with confidentiality.

### Data Analysis

The processing of missing data to avoid biases from loss was carried out through the multiple allocation method (Hair et al., 2018). Ten bases imputed by chained equations with 10 iterations were obtained. In order to test the difference between hierarchically clustered models, the method by Li et al. (1991) was used for the data imputed.

The parental stress models were adjusted by using least square multiple linear regression, proving in all models the assumption of linearity, residual normality, homoscedasticity, and presence of outliers. In the case of parental satisfaction, beta regression was used through R package betareg (Cribari-Neto and Zeileis, 2010), having previously normalised the scores in the 0–1 range; the residuals were checked to determine the model adjustment. For all models, collinearity analysis was performed, verifying tolerance and VIF for each coefficient, with no multicollinearity being found.

The previous analysis of the data regarding parental satisfaction identified the existence of three atypical cases that had residual values very different from the total sample affecting the fit of the model, so it was decided to remove them from the final analysis. Statistical software R 3.6 was used.

## Results

### Descriptive Analysis

Table 1 shows the descriptive values of the scales applied. Between mothers and fathers, higher statistically significant values were observed in mothers in parental stress and satisfaction.

**Table 1:** Descriptive scales.

**Variables**	**Total**					**Father**			**Mother**					
	**n**	**M**	**SD**	**min**.	**max**.	**n**	**M**	**SD**	**n**	**M**	**SD**	**t**	p^*^	**d**
Parental satisfaction	244	4.757	0.647	1	5	123	4.619	0.852	121	4.898	0.265	*t*(145.7) = 3.47	0.001^**^	0.44
Parental stress	244	2.368	1.018	1	5	123	2.058	0.878	121	2.683	1.056	*t*(232.8) = 5.02	0.001^**^	0.64
Life satisfaction	244	5.484	1.099	1	7	123	5.434	1.192	121	5.534	0.998	*t*(236.0) = 0.71	0.479	0.09
Work-Home conflict^a^	174	0.711	0.655	0	3	105	0.665	0.572	69	0.782	0.763	*t*(117.1) = 1.09	0.280	0.21
Home-Work conflict	174	0.325	0.497	0	3	105	0.310	0.472	69	0.348	0.534	*t*(132.9) = 0.48	0.629	0.09
Perceived social support	244	4.408	0.879	1	5	123	4.350	0.904	121	4.468	0.852	*t*(241.5) = 1.06	0.292	0.14

*The averages of the items in each scale are presented*.

a *The Work-Home Questionnaire was only applied to the participants that had a remunerated job*.

* *p < 0.05;*

** *p < 0.01*.

### Parental Stress Scale Analysis

A confirmatory factor analysis of the *Parental Stress Scale* (Oronoz et al., 2007) was performed with the first sample (352 parents with children between 2 to 12 years of age). The adjustment of the solution obtained in the Spanish sample was modelled, where a factor of *parental satisfaction* made up of items 1, 3, 4, 11, and 12; and a factor of *parental stress*, composed of items 2, 5, 6, 7, 8, 9, and 10 were distinguished, as shown in Table 2.

**Table 2:** Parental Stress Scale.

**Items**
1. I am happy in my role as a parent (PS)^a^
2. Caring for my child sometimes takes away more time and energy than I have (PSt)^b^
3. I feel very close to my child (PS)
4. I enjoy spending time with my child (PS)
5. The biggest source of stress in my life is my child (PS)
6. Having a child leaves little time and flexibility in my life (PSt)
7. Having a child has been a financial burden (PSt)
8. I find it difficult to balance different responsibilities because of my child (PSt)
9. My child's behaviour is often uncomfortable or stressful (PSt)
10. I am overwhelmed (with too much pressure), because of the responsibility of being a parent (PSt)
11. I am satisfied as a parent (PS)
12. I enjoy my child (PS)

*Parental Stress Scale (Oronoz et al., 2007) corresponds to the Spanish adaptation of the Parental Stress Scale-PSS (Berry and Jones, 1995)*.

a *PS, Parental Satisfaction*.

b *PSt, Parental Stress*.

The MLR estimation (robust model) was used for the CFA, considering the items as continuous variables. The adjustment indexes obtained were: χ^2^(53) = 79.159, *p* <.001; RMSEA = 0.037 (0.024–0.050); CFI = 0.968; TLI = 0.961, values that can be considered satisfactory. It was not considered appropriate to propose any re-specification to the model. Both subscales, parental satisfaction and stress, showed adequate internal consistency, with a coefficient omega of 0.88, CI 95% [0.81, 0.94] and 0.85, CI 95% [0.82, 0.87], respectively. The correlation between parental stress and parental satisfaction factors was very small (0.03).

### Regression Analysis

First, a multiple regression analysis was performed with parental stress and satisfaction as dependent variables. The evaluation of the models was carried out through two procedures: comparison of predictive capacity through Anovas and comparison of variation with the coefficient of determination of *R*^2^. The analysis looked at two groups of variables, in addition to the parent's gender: demographic control variables (socioeconomic status, parent's age, number of children, children's age, and partnership status); and psychosocial predictor variables (work-home conflict, home-work conflict, perceived social support, and satisfaction with life). Gender moderation was also assessed regarding each of the variables considered.

The first analysis was performed on parental stress with a hierarchical multiple regression with five models. Model 1 analysed the predictive capacity of control variables (*R*^2^ = 0.16). Model 2 examined whether there was gender moderation of the relationship between control variables and parental stress (*R*^2^ = 0.18). Psychosocial predictor variables (*R*^2^ = 0.37) were incorporated into the analysis of model 3. Model 4 adds gender moderation to the relationship between psychosocial predictor variables and parental stress (*R*^2^ = 0.38). Considering that there was no gender moderation with respect to control variables or psychosocial predictors, model 5 was tested, which included only gender, control, and psychosocial variables without any moderation (*R*^2^ = 0.34). The results of the regression are presented in Table 3.

**Table 3:** Hierarchical multiple regression analysis of parental stress.

**Variables**	**Model 1**		**Model 2**		**Model 3**		**Model 4**		**Model 5**	
	**β**	***p***	**β**	***p***	**β**	***p***	**β**	***p***	**β**	***p***
Constant	−0.849	< 0.001	−0.846	< 0.001	−0.585	< 0.001	−0.648	0.017	−0.593	< 0.001
Gender (female)	0.614	< 0.001^**^	0.767	0.069	0.758	0.045^*^	0.874	0.080	0.508	< 0.001^**^
**Control variables**										
SES Ca-Cb^a^	0.347	0.057	0.239	0.450	0.074	0.783	0.099	0.723	0.229	0.175
SES D-E	0.44	0.029^*^	0.287	0.378	−0.169	0.570	−0.09	0.777	0.109	0.573
Parent's age 1^b^	−0.022	0.057	−0.033	0.056	−0.043	0.007^**^	−0.039	0.015^*^	−0.022	0.037^*^
Parent's age 2	0.002	0.111	0.003	0.148	0.004	0.067	0.004	0.053	0.003	0.043^*^
N° Children (2)	0.382	0.052	0.237	0.366	0.313	0.184	0.31	0.190	0.373	0.040^*^
N° Children (3 or more)	0.462	0.114	0.291	0.443	0.116	0.736	0.15	0.668	0.31	0.243
Child's age (school only)	0.074	0.642	0.327	0.166	0.527	0.016^*^	0.494	0.025^*^	0.175	0.238
Child's age (school and preschool)	−0.154	0.520	0.181	0.603	0.389	0.220	0.318	0.321	−0.042	0.849
Partnership status (with partner_other)	0.145	0.435	0.073	0.790	−0.179	0.468	−0.136	0.586	−0.069	0.702
Partnership status (no partner)	−0.052	0.716	−0.078	0.701	−0.28	0.159	−0.178	0.397	−0.276	0.060
**Interaction**										
Gender x SES Ca-Cb			0.117	0.766	0.143	0.679	0.107	0.765		
Gender x SES D-E			0.281	0.513	0.485	0.225	0.378	0.372		
Gender x parent's age 1			0.024	0.323	0.044	0.057	0.046	0.048^*^		
Gender x parent's age 2			−0.003	0.396	−0.003	0.236	−0.004	0.192		
Gender x N° Children (2)			0.346	0.394	0.16	0.661	0.081	0.828		
Gender x N° Children (3 or more)			0.291	0.647	0.251	0.665	0.142	0.813		
Gender x Child's age (school only)			−0.511	0.125	−0.715	0.019^*^	−0.695	0.023^*^		
Gender x Child's age (school and preschool)			−0.732	0.145	−0.841	0.062	−0.678	0.144		
Gender x Partnership status (with partner_other)			0.193	0.623	0.307	0.397	0.26	0.478		
Gender x Partnership status (no partner)			−0.008	0.980	−0.097	0.734	−0.238	0.441		
**Psychosocial variables**										
Satisfaction with life					−0.162	0.021^*^	−0.145	0.123	−0.134	0.051
WHC/Work-Home^c^					0.168	0.133	0.243	0.104	0.16	0.124
WHC/Home-Work					0.272	0.040^*^	0.191	0.200	0.266	0.032^*^
Perceived social support					−0.019	0.787	0.055	0.574	−0.015	0.823
**Interaction**										
Gender x Satisfaction with life							−0.051	0.725		
Gender x WHC/Work-Home							−0.182	0.377		
Gender x WHC/Home-Work							0.172	0.437		
Gender x Perceived social support							−0.176	0.222		
*R*^2^	0.16		0.18		0.37		0.38		0.34	
Δ*R*^2^		0.001^**^	0.02	0.916	0.19	0.001^**^	0.01	0.667	−0.04	0.577

*The constant represents a male of very high socioeconomic status, with one preschool child and in a partnership (married/civil union/de facto relationship). All numerical variables are standardised*.

a *SES Ca-Cb, upper-middle and middle socioeconomic status, SES D-E, middle-lower and low socioeconomic status*.

b *Parent's age 1 = linear effect, Parent's age 2 = quadratic effect*.

c *WHC/Work-Home = work-home conflict, negative interaction subscale work-home; WHC/Home = work-home conflict, negative home-work negative interaction subscale*.

* *p < 0.05;*

** *p < 0.01*.

Model 5 was kept because, while it shows a slight decrease in its predictive capacity compared to model 3 (Δ*R*^2^ = −0.03), it also shows an adequate fit with fewer predictor variables, being a more parsimonious model. The significant coefficients of the model are: being female (it was observed that mothers have on average higher levels of parental stress than fathers); parents age; having two children, and home-work conflict. As observed in models 2 and 4, there was no moderating effect of the parent's gender on the relationship between parental stress and control and predictor variables. One aspect that is of interest is the increase in the predictive capacity of model 3 (Δ*R*^2^ = 0.19) after the incorporation of psychosocial variables.

**Figure 1:**
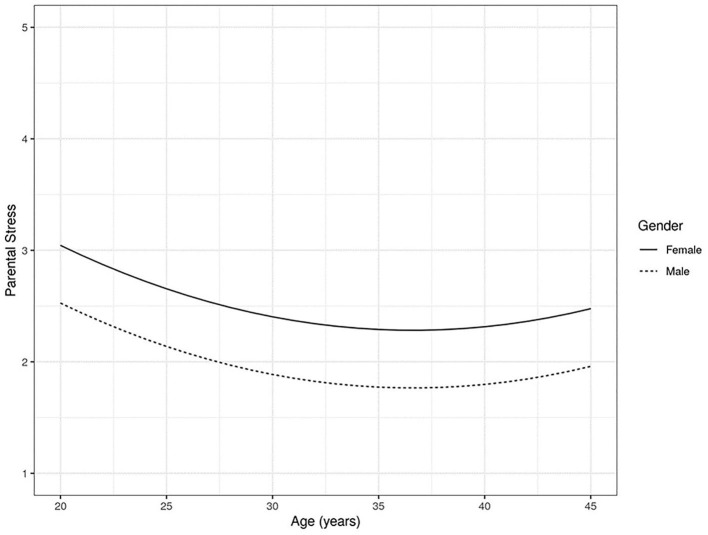
Parental stress and parent's age relationship.

A quadratic effect of the parent's age was observed. Figure 1 shows a U-effect of the parent's age on parental stress. Young parents (23 years of age with a mean of 2.3) have the highest levels of stress; and then, as the parent's age increases, there is a decrease in their stress level, reaching a minimum point of 1.78 at 36.6 years of age; and then it increases again as the parent's age rises, though without reaching the levels of young parents, showing a maximum level of 1.97 at 45 years of age.

The second regression was carried out with regard to parental satisfaction following the same modelling for parental stress analysis. The multiple regression model presented adjustment problems for non-compliance with the assumptions of linearity and homoscedasticity, mainly because of the presence of the ceiling effect on the scale due to the high concentration of high values. In response, a beta regression was performed, which is useful when the criterion variable is continuous and it is restricted to the range 0–1 (Ferrari and Cribari-Neto, 2004). This regression model allows modelling different distribution types, being an alternative analysis for non-linear relations with a minimum and maximum specified in advance (Salinas-Rodríguez et al., 2006).

Model 1 analysed the predictive capacity of control variables (pseudo *R*^2^ = 0.20). Model 2 examined gender moderation in the relation between control variables and parental satisfaction (pseudo *R*^2^ = 0.24). Model 3 incorporated the psychosocial predictor variables (pseudo *R*^2^ = 0.28). In model 4, gender moderation was added with respect to psychosocial predictor variables (pseudo *R*^2^ = 0.29), which did not turn out to be not significant for this model; however, there is a statistically significant moderation effect of gender with respect to the coefficient of partnership status (no partner). Finally, model 5 was tested, which only differs from the previous one by the incorporation of the gender moderation variable with respect to the partnership status coefficient (no partner) and, although a slight decrease in its predictive capacity is observed with a pseudo *R*^2^ = 0.26, there is no statistically significant difference from model 4. The results of regression for each model are presented in Table 4.

**Table 4:** Beta regression analysis of parental satisfaction.

**Variables**	**Model 1**		**Model 2**		**Model 3**		**Model 4**		**Model 5**	
	**β**	***p***	**β**	***p***	**β**	***p***	**β**	***p***	**β**	***p***
Constant	2.879	< 0.001	2.89	< 0.001	2, 781	0.017	2, 853	0.056	2, 859	0.004
Gender (female)	0.14	0.313	0.139	0.752	0.155	0.725	0.08	0.727	0.018	0.923
**Control variables**										
SES Ca-Cb^a^	0.154	0.421	0.295	0.342	0.414	0.189	0.386	0.228	0.221	0.263
SES D-E	0.058	0.786	0.135	0.675	0.358	0.287	0.293	0.405	0.2	0.368
Parent's age 1^b^	0.008	0.517	−0.007	0.710	−0.006	0.767	−0.006	0.758	0.003	0.840
Parent's age 2	−0.001	0.587	−0.001	0.804	0	0.949	0	0.949	−0.001	0.727
N° Children (2)	−0.249	0.246	−0.558	0.048^*^	−0.652	0.021^*^	−0.68	0.016^*^	−0.304	0.159
N° Children (3 or more)	−0.149	0.642	−0.362	0.374	−0.306	0.454	−0.374	0.366	−0.201	0.534
Child's age (school only)	0.156	0.371	0.254	0.314	0.164	0.520	0.185	0.475	0.182	0.299
Child's age (school and preschool)	0.406	0.121	0.885	0.018^*^	0.801	0.034^*^	0.836	0.029	0.478	0.072
Partnership status (with partner_other)	−0.306	0.132	−0.203	0.488	−0.135	0.648	−0.192	0.524	−0.12	0.679
Partnership status (no partner)	−0.471	0.003^**^	−0.874	< 0.001^**^	−0.788	0.001^**^	−0.822	0.001^**^	−0.704	0.003^**^
**Interaction**										
Gender x SES Ca-Cb			−0.265	0.510	−0.296	0.462	−0.27	0.513		
Gender x SES D-E			−0.138	0.756	−0.26	0.561	−0.162	0.731		
Gender x parent's age 1			0.021	0.418	0.013	0.627	0.013	0.633		
Gender x parent's age 2			0	0.973	0	0.901	0	0.913		
Gender x N° Children (2)			0.573	0.189	0.718	0.103	0.747	0.095		
Gender x N° Children (3 or more)			0.293	0.668	0.265	0.702	0.388	0.581		
Gender x Child's age (school only)			−0.102	0.776	−0.008	0.983	−0.031	0.932		
Gender x Child's age (school and preschool)			−0.689	0.203	−0.656	0.226	−0.679	0.222		
Gender x Partnership status (with partner_other)			−0.192	0.650	−0.272	0.523	−0.213	0.622	−0.341	0.404
Gender x Partnership status (no partner)			0.717	0.028^*^	0.698	0.035^*^	0.746	0.037^*^	0.568	0.070
**Psychosocial variables**										
Satisfaction with life					0.137	0.083	0.145	0.177	0.126	0.105
WHC/Work-Home^c^					0.01	0.927	0.075	0.653	−0.002	0.985
WHC/Home-Work					−0.007	0.957	0.038	0.815	−0.001	1.000
Perceived Social Support					0.099	0.244	0.088	0.447	0.095	0.246
**Interaction**										
Gender x Satisfaction with life							−0.017	0.918		
Gender x WHC/Work-Home							−0.102	0.637		
Gender x WHC/Home-Work							−0.109	0.632		
Gender x Perceived social support							−0.018	0.914		
(phi)	3.726	< 0.001	3.93	< 0.001	4.117	< 0.001	4.169	< 0.001	3.996	< 0.001
Pseudo *R*^2^	0.20		0.24		0.28		0.29		0.26	
Pseudo Δ*R*^2^		0.057	0.04	0.534	0.04	0.243	0.01	0.830	0.02	0.939

*The constant represents a male of very high socioeconomic status, with one preschool child and in a partnership (married/civil union/de facto relationship). All numerical variables are standardised*.

a *SES Ca-Cb, upper-middle and middle socioeconomic status; SES D-E, middle-lower and lower socioeconomic status*.

b *Parent's age 1 = linear effect; Parent's age 2 = quadratic effect*.

c *WHC/Work-Home = work-home conflict, negative interaction subscale work-home; WHC/Home = work-home conflict, negative home-work negative interaction subscale*.

* *p < 0.05;*

** *p < 0.01*.

Model 5 only presents as statistically significant the coefficient of partnership status (no partner), result that is constant in all models. Parents with no partner show lower levels of parental satisfaction compared to their peers in a partnership (married, de facto relationship, and civil union status). In this regard, the gender moderation effect of the parent with respect to the partnership status coefficient in model 4 is of interest, establishing that mothers with no partner would have higher levels of parental satisfaction compared to fathers in the same condition.

#### Dominance Analysis

Finally, a dominance analysis was performed (Budescu, 1993). This analysis is a useful complement to multiple regression analyses because it allows to estimate the relative significance of predictors without the limitations presented by common measurements such as standardised beta values or partial or semi-partial correlations (Tonidandel and Lebreton, 2011). General and conditional analysis were considered in the dominance analysis. The conditional dominance analysis compares the input of each predictor in models with a specific number of additional predictors (Azen and Budescu, 2003). The general dominance analysis is the average of the conditional dominances of each predictor and, in linear models, it corresponds to the square semi-partial correlation.

The dominance analysis of the results of the regression of the variables stress and parental satisfaction, considered the variables gender, satisfaction with life, work-home conflict, home-work conflict, and social support individually and the other control variables in block (socioeconomic status, parent's age, number of children, children's age, and partnership status), which were called *sociodemographic factors*.

**Figure 2:**
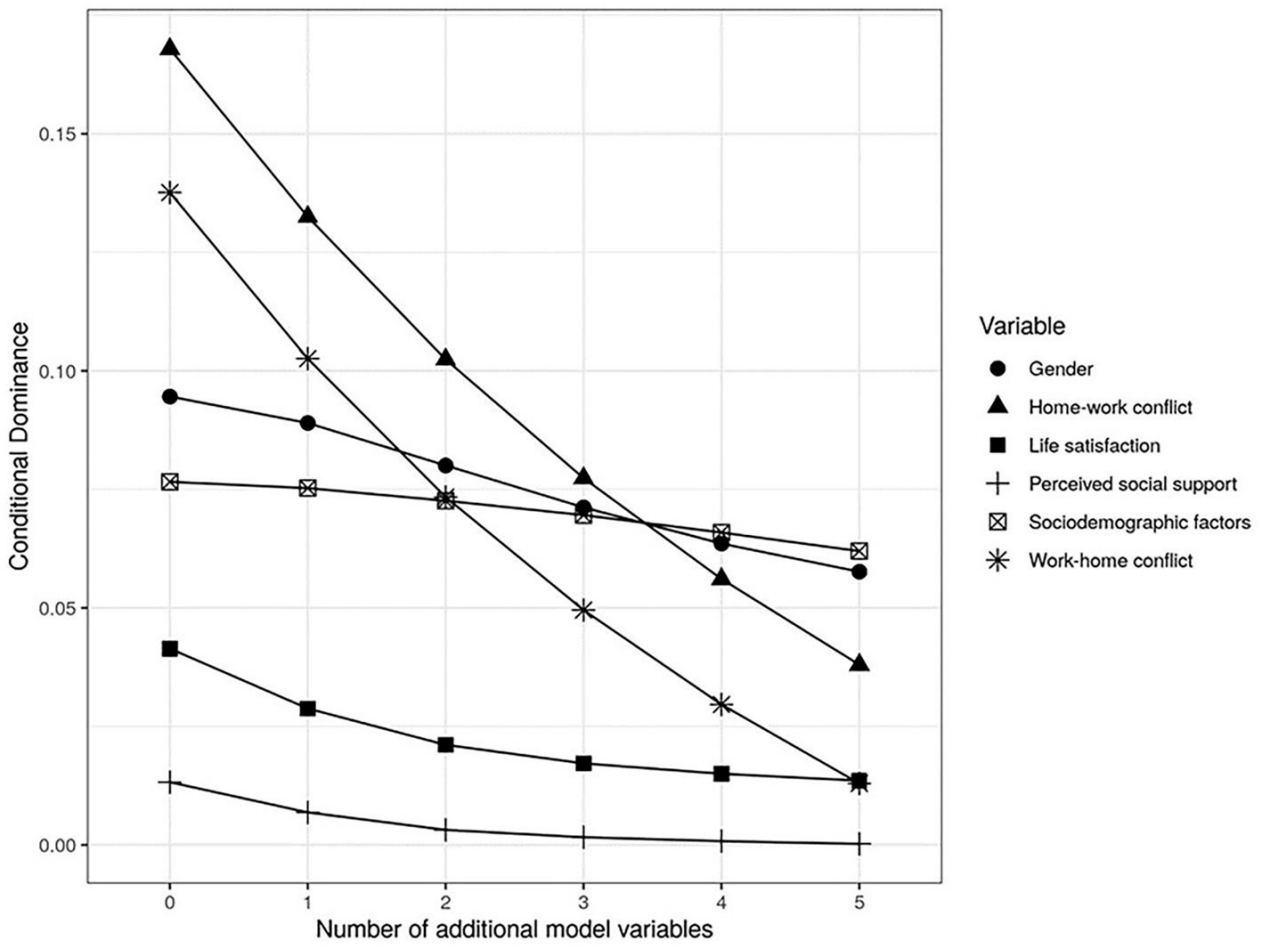
Conditional dominance analysis of parental stress with control variables and psychosocial predictors.

Regarding the results of the regression of Model 5 of parental stress, the conditional dominance analysis shows that the predictive capacity of the home-work conflict decreases substantially as the other variables are added to the model. Similar behaviour is observed in the work-home conflict. In turn, the conditional dominance of sociodemographic factors and gender is almost unaffected, which evidences that its predictive capacity is independent of the rest of the predictors (see Figure 2).

On the other hand, the general dominance analysis showed that the predictor that has the highest mean contribution to parental stress is home-work conflict with an *R*^2^ close to 0.10. The remaining predictors in order of mean contribution were: gender (*R*^2^ = 0.076), sociodemographic factors block (*R*^2^ = 0.07), work-home conflict (*R*^2^ = 0.068), satisfaction with life (*R*^2^ = 0.023), and perceived social support (*R*^2^ = 0.004).

**Figure 3:**
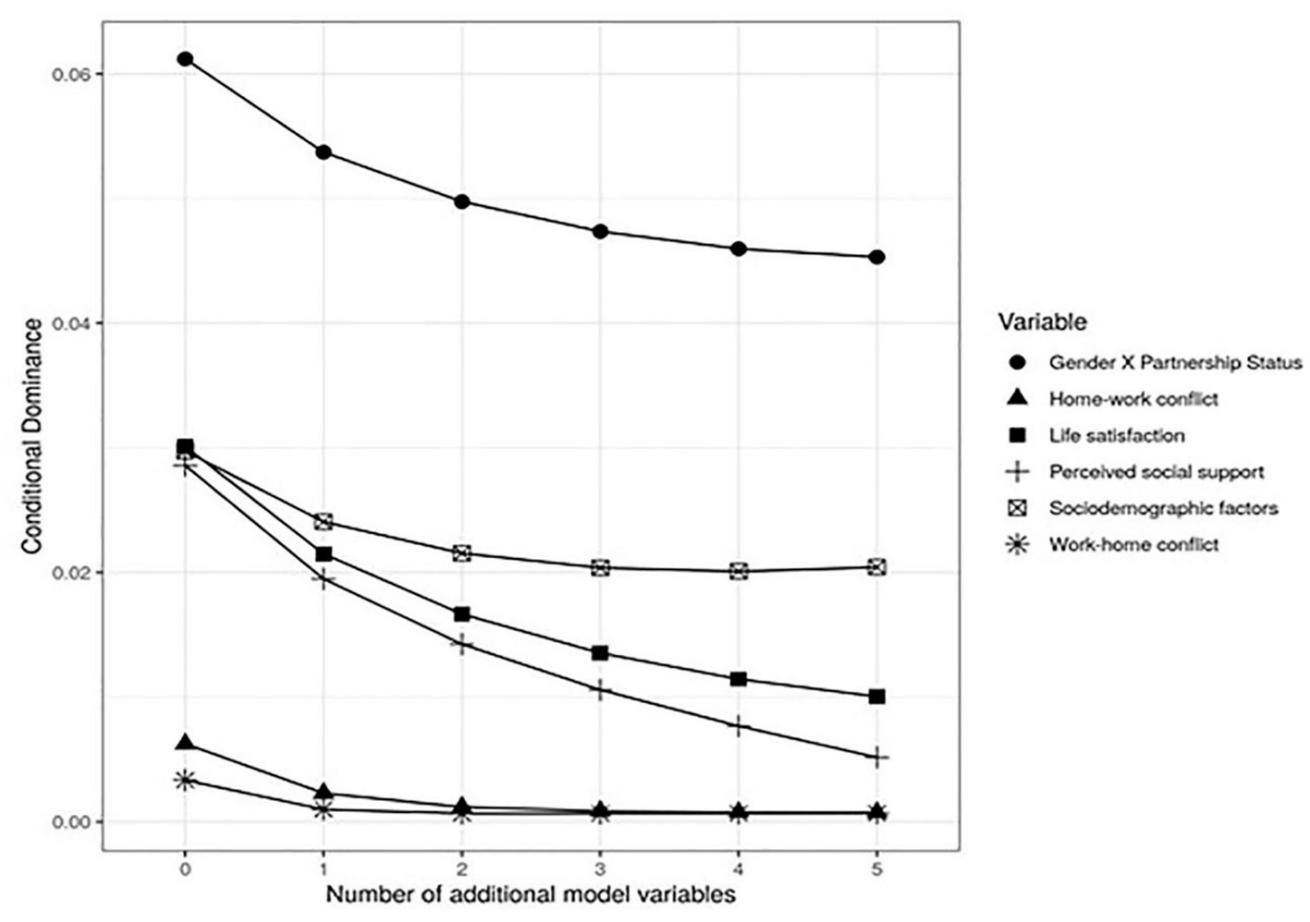
Conditional dominance analysis of parental satisfaction with control variables, psychosocial predictors, and gender x partnership status moderation.

The conditional dominance analysis of the parental satisfaction regression carried out based on model 5 composed of the control variables, psychosocial predictors, and the moderation of gender (female) with respect to partnership status (which included the variable gender as a block) shows that the moderation of gender with respect to the partnership status is the predictor that presents a greater individual contribution. The contribution of this predictor is almost independent of the rest of the variables of the model, showing a stability in its dominance. Similar behaviour is observed in sociodemographic factors, although their individual contribution is lower. On the contrary, the conditional dominance of satisfaction with life and social support decreases significantly as the other variables of the model are added, as shown in Figure 3.

The general dominance analysis shows that the predictor that provides the greatest mean contribution to the variance of parental satisfaction is moderation of gender with respect to partnership status, with an *R*^2^ = 0.050, which is consistent with the result of the conditional dominance analysis. The remaining predictors in order of mean contribution were: sociodemographic factors block (*R*^2^ = 0.023), satisfaction with life (*R*^2^ = 0.017), perceived social support (*R*^2^ = 0.014), home-work conflict (*R*^2^ = 0.002), and work-home conflict (*R*^2^ = 0.001).

## Discussion

The aim of this research was to analyse the stress and parental satisfaction of parents in order to establish the relationship of these two poles of parental experience with the work-home and home-work conflict, perceived social support, satisfaction with life. As covariates, and with the fundamental purpose of avoiding their possible confusing role, the effect of socioeconomic status, partnership status, parents' age, and number of children were considered. In all the analyses, not only was the parent's gender included, but it was also examined whether there were gender moderating effects for each variable considered.

To measure parental stress and satisfaction, the Parental Stress Scale- PSS (Berry and Jones, 1995), in its Spanish version (Oronoz et al., 2007) was used. The confirmatory factor analysis showed adequate adjustment indexes for this 12-item and two-factor version (baby rewards, called parental satisfaction in this study; and parental stress). Almost no relationship between stressors and parental satisfaction was observed. Although a relative autonomy of both factors can be expected, the lack of correlation is striking. Other studies have observed the existence of a moderate negative relationship between parental satisfaction and stress, showing that parents who are more satisfied with their parental role tend to present lower levels of parental stress (Pérez et al., 2010; Ribeiro et al., 2013). The results observed show a clear separation between the evaluative processes of perceived stress associated with the parental role (a content more focused on the immediate present experience) and parental satisfaction, which refers to the more global significance of the experience of being a parent, regardless of the effort involved.

In the total sample, the stress level was moderate, but the level of satisfaction was very high. The very high scores, with little dispersion, of parental satisfaction suggest that social desirability plays an important role in the assessment of this aspect.

Another indicator of the independence of parental stress from parental satisfaction is the gender differences observed. Mothers presented higher levels of parental stress and satisfaction than fathers. In general, studies show higher levels of parental stress in mothers than in fathers; on the other hand, gender differences with respect to parental satisfaction do not show a consistent pattern (Salonen et al., 2010; Lozano et al., 2016; Pérez and Santelices, 2016; Pereira et al., 2017). In the multivariate analyses, gender was only related to the greatest stress in mothers and did not show to be a predictor of greater satisfaction.

Four variables were statistically significant to predict parental stress: being female, having two children, home-work conflict, and parent's age. Except for parent's age, the relationship with parental stress was positive.

The relevance of the predictive capacity of gender regarding parental stress, specifically being female, is probably accounted for by the persistence of the gender division of labour that assigns women a leading role in raising children despite transformations in current family arrangements, which implies more time devoted to tasks associated to the care of children and domestic chores (Bidegain and Calderón, 2018; Nomaguchi and Milkie, 2020). Various studies confirm that mothers are more exhausted, more stressed, and have lower levels of subjective well-being than fathers (Connelly and Kimmel, 2015; Musick et al., 2016; Nomaguchi and Milkie, 2020).

Male chauvinism is one of the dominant traits of masculinity in Latin America up to the present (Fuller, 2018). In Chile, the predominant role of the mother in upbringing, from colonial times to the present day, has been highlighted by various authors (Montecino, 2018). With industrial capitalism, a very strong patriarchal normative family model is installed where women take over the care of the children and the house chores, and men are the providers and authority (Olavarría, 2014). Nowadays, in spite of the crisis in the order of traditional gender because of cultural changes, there still would be a masculine social contract founded on the sexual division of labour (Valdés, 2020), with greater social demands towards mothers regarding the care of their children as well as the practical and affective spheres (Araujo and Martuccelli, 2012).

The increase in parental stress in parents with more than one child can be expected due to the number of demands, especially considering that the sample was limited to families with children under 12 years of age. The effect was only statistically significant in families with two children and not with three or more, which is probably attributable to the low number of families in the latter condition (only 7%). Research tends to show greater stress in parents with more children. It must be considered that the traditional support role in raising older siblings has been decreasing (Flouri, 2005; Pérez et al., 2010).

The relationship observed between parental stress and work-home conflict is consistent with the perception of various researchers that this is becoming one of the main stressors of current parenting. Current social practises make parenting more demanding as they imply: more limited support networks for upbringing, fewer possibilities for autonomous life and displacement of children in current cities, more expectations regarding parental roles, greater demands of parents presence in upbringing, all of which comes into conflict with characteristics proper of the present working world (Pérez et al., 2010; Lozano et al., 2016). This conflict is exacerbated by the growing participation of women in labour and social life, and the lag in the adjustment of the forms of social relationship to this process (Avolio and Di Laura, 2017). Contrary to the traditional approach to the work-home conflict, the direction presented in this study is of interest, highlighting that only the subscale of home-work conflict was significant. This suggests that the perceived stress derives from the parents' dedication to the multiple activities and responsibilities that family life and caring for their children imply, which interfere with working life (Jain and Nair, 2013).

An unexpected finding regarding parental stress was the non-linear effect, specifically the quadratic effect (U-effect) of the parent's age. Young parents are the ones who show the highest levels of parental stress, which then decreases as the age of the parents increases, presenting a turning point at 36.6 years of age, where stress begins to increase again as the parent's age rises but which does not reach the levels of stress in young parents.

A higher level of parental stress in young parents is a consistent finding of research (Pérez and Santelices, 2016; Tacca et al., 2020). Probably, in the current sociocultural context, having children at an early age implies additional stress derived from the fewer resources available for their care and the conflict with other demands of this vital stage, in addition to the lower experience in assuming responsibilities of that nature (Oronoz et al., 2007; Bornstein, 2015; Pérez and Santelices, 2016; Nelson-Coffey et al., 2019). In older parents, it is less clear which factors could account for the greater stress observed. As a hypothesis, it would be possible to posit that it could derive from the conjunction of parental demands with others proper of that later stage in the life cycle. Given that, in the sample studied, it is more frequent for older parents to have children reaching adolescence, it is tempting to hypothesise that this would be accounted for by the greater demands of this vital moment of their children; however, the effect of age on the children has been controlled in the analysis, so it could not be a plausible explanation, at least not directly.

Although the dominance analysis regarding parental stress indicated that the home-work conflict is the predictor with the highest average contribution regarding parental stress (general dominance), its conditional dominance decreased as the other predictors were incorporated. Gender and the sociodemographic factors block are the predictors that conditionally dominate the others in the models that include all the predictors, showing that they have an independent predictive capacity with respect to the other variables of the model.

Regarding parental satisfaction, only the partnership status was a significant predictor, with a negative relationship between the category “no partner” and parental satisfaction being recognised. In this regard, a moderation effect was observed between gender and partnership status, indicating that mothers with no partner showed greater satisfaction than fathers with no partner. Although in the final model this interaction did not show statistical significance, it did in the various previous models. Likewise, in both the general and conditional dominance analysis, this moderation was the most relevant predictor. These results show that parental satisfaction in men with no partner is decreased. It would be interesting to explore whether it is the partnership status or the possibility of daily contact with the children derived from coexistence that can account for this result.

Despite its relevance, this study did not show an effect of satisfaction with life or perceived social support neither on parental stress nor parental satisfaction. It was hypothesised that satisfaction with life could capture an individual dimension of life perception that could influence the perception of stress and parental satisfaction, but no greater contribution of this factor was observed. It is interesting to note that this have occurred even in relation to parental satisfaction, indicating that both valuations have different roots. Probably, the not very central role of perceived social support is related to the fact that a very general measurement of it was used and not of social support that is more directly related to the exercise of parenting. In addition, not using the survey in its entirety might have limited its measurement.

The results obtained are of interest to understand the effects of the accelerated transformations in social and family life over the past decades and their impact on the experience of parenting. In particular, it highlights the need to explore in a differentiated way the aspects related to experiences of stress and oppression that parenting may be implying in the context of current social life for certain subgroups of parents, particularly for mothers, on the one hand; and, on the other hand, the satisfaction with which the maternal or paternal condition is experienced, despite the above. Various authors have been highlighting how excessively idealised views of the maternal or paternal condition make it impossible to understand this dimension of human life in all its complexity, identifying both its rewarding aspects and its costs (Nomaguchi and Milkie, 2003; Umberson et al., 2010). This is of particular interest in a social context where social mandates regarding what being a parent implies have increased, placing increasingly demanding expectations of attention and care for children while, on many occasions, the social supports for the exercise of the parental role decrease (Murray, 2015; Vergara Del Solar et al., 2018).

A challenge for further research is the study of parental satisfaction. This construct has been little explored in itself, being subsumed in the sphere of the perception of parental competence or the overall well-being and satisfaction of those who are parents (Menéndez et al., 2011; Gilmore and Cuskelly, 2012). However, its study poses relevant challenges. The results obtained suggest that the scale shows ceiling effects that limit its discriminatory capacity. It is not clear whether a factor of social desirability could be contributing to this.

This study focused on parents with children between the ages of 2 and 12. The exercise of parenting with younger children implies specificities, so they were not considered in the sample. Neither were parents of adolescents or those with adult children included. One of the challenges for future research is a broad life-span look at parenting. So far, most of the studies have focused on the transition to parenting and the experience with minor children (Nomaguchi and Milkie, 2020). However, focusing on this age range of the children makes these findings less generalisable, and they are considered a limitation of the study.

The findings obtained are of interest for the study of parenting from the experience of being a father or a mother. It is relevant to delve into the positive aspects of this experience such as the attributions of meaning or the feelings of joy, pride, gratitude, and satisfaction; as well as the demands and conflicts associated with this very central dimension of many people's lives.

## Data Availability Statement

The raw data supporting the conclusions of this article will be made available by the authors, without undue reservation.

## Ethics Statement

The studies involving human participants were reviewed and approved by Comité de Etica de Universidad de Concepción. The participants provided their written informed consent to participate in this study.

## Author Contributions

MO is first authorship. FC and CB have contributed equally to this work and share second authorship. All authors contributed to the article and approved the submitted version.

## Conflict of Interest

The authors declare that the research was conducted in the absence of any commercial or financial relationships that could be construed as a potential conflict of interest.
